# Stand structure and plant diversity characteristics of typical artificial forests after natural recovery in the hilly region of central Hainan

**DOI:** 10.3389/fpls.2025.1629250

**Published:** 2025-07-08

**Authors:** Jianxing Wei, Haihui Chen, Xuebiao Yu, Zhaobin Guo, Xuefeng Zhang, Leyu Tian, Shouqian Nong

**Affiliations:** ^1^ Hainan Academy of Forestry (Hainan Academy of Mangrove), Haikou, China; ^2^ Hainan Wenchang Forest Ecosystem Observation and Research Station, Wenchang, China; ^3^ School of Tropical Agriculture and Forestry, Hainan University, Haikou, Hainan, China

**Keywords:** artificial forest transformation, natural succession, tropical artificial forest, plant diversity, stand structure

## Abstract

**Introduction:**

The global expansion of artificial forests has highlighted the necessity of restoring their ecological service functions and understanding natural succession mechanisms in forest restoration ecology. However, comprehensive analyses of community assembly in tropical artificial forests following long-term natural recovery and their divergence from zonal vegetation remain insufficient.

**Methods:**

In this study, the stand structure and plant diversity were investigated in three typical tropical artificial forests (*Acacia mangium, Hevea brasiliensis*, and *Eucalyptus*) after 20 years of natural recovery, alongside 33-year-old natural secondary forests, in the Fengmu Experimental Forest Farm, Hainan Province. The relationships between plant diversity and community structural factors in artificial forests were also examined.

**Results:**

The findings can be summarized as follows. (1) *Acacia mangium* forests exhibited superior natural regeneration, whereas the naturally regenerated trees in all plantations displayed significantly smaller mean diameter at breast height and height than those in the natural secondary forests. (2) Although the species diversity in certain forest layers of plantations approached that of natural secondary forests, notable differences persisted, and woody plants in plantations lacked the phylogenetic traits observed in natural secondary forests. (3) Redundancy analysis showed that the greater densities and canopy cover of planted trees inhibited arbor layer diversity but promoted phylogenetic dispersion. High tree density facilitated shrub layer establishment, whereas height growth in regenerated trees and shrubs inhibited shrub diversity through resource competition. Additionally, the increased diameter class variation in regenerated trees and taller shrub-herb layers reduced herb layer diversity due to resource limitations.

**Discussion:**

After 20 years of natural recovery, plantations have developed multi-aged, vertically stratified mixed stands. However, growth constraints on woody plants and limited biodiversity recovery persist. Structural optimization is crucial for enhancing niche differentiation and accelerating succession toward climax forest communities.

## 1 Introduction

Since the mid-20th century, artificial forests have expanded globally with a decline in natural forest areas. Between 1990 and 2020, artificial forests increased by 123 million hectares, reaching 2.94 × 10^9^ ha in 2020 and accounting for 7.24% of the global forest area (Food and Agriculture Organization (FAO), 2020). Although artificial forests meet timber demands, they also contribute to certain ecological problems such as diminished soil and water conservation, biodiversity loss, and ecosystem fragility (Xie et al., 2020). In ecologically sensitive regions and biodiversity hotspots, the restoration of natural forests is essential for conservation and sustainable management (Payn et al., 2015; Silva et al., 2019).

As a designated ecological pilot zone in China and a global biodiversity hotspot, Hainan Province faces significant challenges in sustainable forest management, which is crucial for biodiversity conservation and ecological security (Chen et al., 2021; Long et al., 2021). Its ecological public welfare forest area covers 869,600 ha, over 20% of which consists of artificial forests concentrated in the central mountainous region. Within the Hainan Tropical Rainforest National Park, 824 km^2^ of artificial forests (19.3% of the park area) are primarily composed of exotic species, such as *Hevea brasiliensis* (HB), *Eucalyptus* (ER), and *Acacia mangium* (AM). Human disturbances, including logging, land reclamation, and intensive afforestation, have resulted in monocultures, simplified stand structures, reduced biodiversity, and weakened ecological functions (Chen et al., 2021; Xue et al., 2015). In parks, the prevalence of artificial forests has intensified landscape fragmentation and habitat degradation, posing a threat to ecosystem integrity. Restoration of these forests to natural conditions is urgently needed to support tropical rainforest recovery, enhance carbon sequestration, and fulfill the park’s ecological protection objectives. Currently, research on plantation restoration in Hainan Tropical Rainforest National Park primarily focuses on changes in vegetation characteristics in single plantation types undergoing natural restoration and on models for restoration transformation (Wang et al., 2025; Du et al., 2025). However, there is a lack of comparative studies examining vegetation characteristics across different plantation types under natural restoration as well as analyses of key stand structure factors essential for the restoration of plant diversity.

Global research on the conversion of artificial forests into natural forests has progressed with proposed approaches including clear-cutting, assisted natural regeneration, and unassisted natural recovery (Ammer et al., 2002; Brunner, 2001; Crouzeilles et al., 2017; Spiecker et al., 2004). Among these, natural recovery is favored in tropical regions owing to its cost-effectiveness and sustainability (Crouzeilles et al., 2017). Long-term natural recovery has been shown to promote native species establishment, development of uneven-aged stand structures, and community convergence with natural forests (Ping et al., 2009; Wang et al., 2021), while correlations between stand structure and plant diversity have gained increasing attention (Li et al., 2023; Sheng et al., 2024). On the other hand, most existing studies have focused on temperate and subtropical forests (Li et al., 2025b; Spiecker et al., 2004; Wang et al., 2021), with limited research on tropical artificial forest recovery and few comparative evaluations across forest types, thereby constraining the development of targeted ecological restoration strategies.

To address the urgent need for artificial-to-natural forest conversion in Hainan’s ecological public welfare forests and the Hainan Tropical Rainforest National Park, this study investigated AM, HB, and ER plantations that underwent 20 years of natural recovery at the Fengmu Experimental Forest Farm, along with a 33-year-old natural secondary forest. This study aimed to address the following questions: (1) What are the stand structure and plant diversity characteristics of tropical artificial forests after two decades of natural recovery? (2) How do these characteristics differ between naturally recovered artificial forests and the zonal vegetation (natural secondary forest)? (3) What is the relationship between plant diversity metrics and key stand structural factors in these artificial forests? These findings can offer a theoretical basis and scientific guidance for promoting artificial-to-natural forest conversion in Hainan’s public welfare forest areas and facilitating the phased removal of artificial forests within the national park.

## 2 Materials and methods

### 2.1 Overview of the study area

The Fengmu Experimental Forest Farm is located in Hainan Province (109°56’E–109°58’E, 19°11’N–19°15’N), which lies in the southwestern part of Tunchang County and spans the transition zone between central Hainan’s mountainous regions and coastal terraces. This hilly region has an average elevation of 235 m and features latosol soil derived from granite. The area experiences a tropical maritime monsoon climate, with an annual mean temperature of 22–27°C, annual precipitation ranging from 1,600 to 2,600 mm, fertile soils, abundant rainfall, ample sunlight, and an average relative humidity exceeding 80%. The forest types in the study area primarily include natural secondary forests and artificial forests, with the latter predominantly composed of AM, HB, and ER (Chen et al., 2024).

### 2.2 Plot establishment and survey

**Figure 1:**
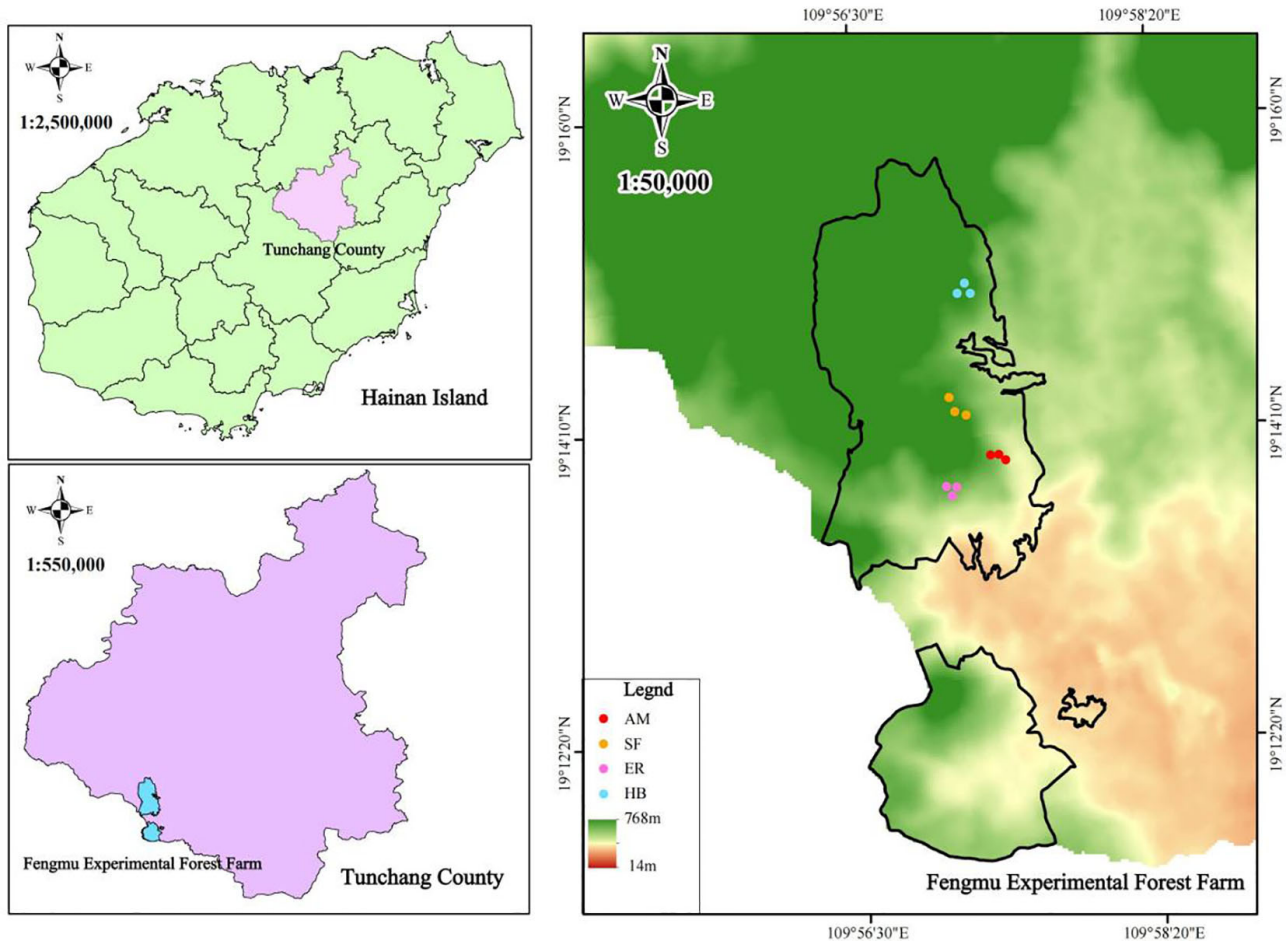
Schematic diagram of the study area and sample plot locations. AM, HB, ER, and SF respectively represent *Acacia mangium* forest, *Hevea brasiliensis* forest, *Eucalyptus* forest, and natural secondary forest.

Based on preliminary reconnaissance and historical plot records, 20-year-old naturally regenerated AM, HB, and ER forests in Hainan’s Fengmu Experimental Forest Farm were selected as research subjects between August and September 2022. A natural secondary forest (SF), developed through natural succession on a *Pinus massoniana* stand logged in 1989, was adopted as a control (**Figure 1**; **Table 1**). In each forest type (AM, HB, ER, and SF), three 20 m × 20 m quadrats were established, each subdivided into four 10 m × 10 m subplots for arbor surveys. At the corners of each quadrat, 5 m × 5 m shrub subplots and 2 m × 2 m herb subplots were set. In the arbor subplots, the species, height, crown width, and branch height were recorded for all woody plants with a diameter at breast height (DBH) ≥ 3 cm. The shrub subplots recorded the species and height of all woody plants (including saplings) with DBH < 3 cm. The herb subplots documented the species, height, and coverage of all herbaceous plants.

**Table 1:** Overview of basic information for each forest stand.

Indicator	Forest stand type			
	AM	HB	ER	SF
Planting year	2000	1998	2000	–
The year of natural enclosure	2002	2002	2002	1989
Natural recovery duration (years)	20	20	20	33
Soil type	Brick red soil	Brick red soil	Brick red soil	Brick red soil

AM, HB, ER, and SF respectively represent *Acacia mangium* forest, *Hevea brasiliensis* forest, *Eucalyptus* forest, and natural secondary forest.

### 2.3 Measurement of plant diversity

Plant species diversity across forest layers in artificial and secondary forests (SF) was evaluated using the Patrick index (R), Shannon index (H), Simpson index (D), and Pielou index (E) (Ma and Liu, 1994), with the corresponding calculation formulas provided below (Equations 1–4).


(1) R = S

(2) $$D=1-\sum_{i=1}^{S}P_{i}^{2}$$

(3) $$H\;=\;-\sum_{i\;=\;1}^{S}\;P_{i}\text{ ln}P_{i}$$

(4) $$E\;=\;\frac{-\sum_{i\;=\;1}^{S}P_{i}\;\text{ ln}P_{i}}{\text{ln}S}$$

where S denotes the total species count within a quadrat, and Pi represents the proportion of individuals of the i-th species relative to the community’s total.

Based on the plant survey data from the research sites, the species in each forest stand were identified using The Plant List database (http://www.theplantlist.org). Species-level phylogenetic trees were constructed for each stand using the “S3” method of the “phylo.maker” function in the V.PhyloMaker2 software package (Jin and Qian, 2019). Faith’s phylogenetic diversity index (PD) was used to assess the phylogenetic α diversity within quadrats (Faith, 1992), and the mean phylogenetic distance (MPD) and mean nearest taxon distance (MNTD) were employed to quantify the phylogenetic β diversity (Webb, 2000). All calculations were performed using the Picante software package.

### 2.4 Measurement of community structural characteristics

The crown coverage, tree density, mean diameter at breast height (DBH), mean height, mean crown width, and branch height of the artificial trees were measured for each plantation. The arbor regeneration layer was defined as woody plants with DBH ≥ 3 cm within the arbor layer, excluding artificial trees.

The arbor regeneration layer, artificial forest shrub layer, and corresponding layers in natural secondary forests were classified into arbor and shrub species based on their life forms, with reference to the Flora of China (https://www.iplant.cn). For each type, the average plant number density and plant height were recorded, along with the overall averages, herb layer coverage, and mean plant height.

The diameter diversity index was utilized to analyze tree size differentiation in the arbor regeneration layer of artificial forests and the arbor layer of natural secondary forests. The DBH-Shannon and DBH-Simpson indices were applied to evaluate the diameter size diversity, while the DBH-Pielou index reflected the evenness of the individual tree size distribution. The corresponding calculation formulas are as follows (Shen et al., 2024) (Equations 5–7):


(5) $$\text{DBH}-\text{Simpson}=1-\sum_{i=1}^{S}P_{i}^{2}$$

(6) $$\text{DBH}-\text{Shannon }=\;-\sum_{i\;=\;1}^{S}P_{i}\text{ ln}P_{i}$$

(7) $$\text{DBH}-\text{Pielou }=\;\frac{-\sum_{i\;=\;1}^{S}P_{i}\text{ ln}P_{i}}{\text{ln}S}$$

where S represents the number of diameter classes of trees in the community, and P_i_ denotes the ratio of the number of trees in the i-th diameter class to the total number of trees across all diameter classes in the community.

### 2.5 Association between plant diversity and community structure elements

To elucidate the influence of community structure on plant diversity in naturally regenerating plantations, redundancy analysis (RDA) was conducted. Plant diversity indices, including the Patrick, Shannon, Simpson, and Pielou indices, as well as PD, MPD, and MNTD, could serve as response variables. The stand structure factors were used as explanatory variables, including canopy cover, tree density, mean DBH, mean height, mean crown width, and branch height of artificial trees; number density, mean height, mean DBH, DBH-Shannon index, DBH-Simpson index, and DBH-Pielou index of the arbor regeneration layer; number density and mean height of the shrub layer; and coverage and mean height of the herb layer.

### 2.6 Data processing

The survey data were compiled and organized using Excel 2019. One-way ANOVA and least significant difference (LSD) multiple comparisons were performed to analyze the stand structure factors and plant diversity across different plantations and SF using SPSS 26.0. The RDA of stand structure and plant diversity in plantations was conducted using Canoco 5.0.

## 3 Results and analysis

### 3.1 Growth overview of different forest stands

As shown in **Table 2**, two decades after natural restoration, distinct growth patterns were observed among forest types. The ER forests exhibited the greatest decline in artificial tree number density (79.50%), resulting in the lowest current density but the tallest mean tree and branch heights. In contrast, AM forests maintained the highest current artificial tree density, with a moderate reduction (52.00%). HB forests showed the highest crown cover, the largest mean DBH, and the smallest decline in density (21.88%). In terms of natural regeneration, the AM forests supported the densest and largest diameter regenerated trees, whereas the ER forests had the tallest. Conversely, HB forests exhibited the smallest DBH and shortest height among the naturally regenerated trees. Notably, the naturally regenerated trees in all plantations had a significantly (P < 0.05) smaller mean DBH compared to those in natural secondary forests. These results highlight the substantial differences in the growth trajectories of artificial and naturally regenerated trees among forest types. Although the AM forests exhibited the most vigorous regeneration (highest density and DBH), natural regeneration in all plantations remained inferior to that in the natural secondary forests.

**Table 2:** Stand growth characteristics of different stands.

Indicator	Forest stand type			
	AM	HB	ER	SF
Canopy coverage rate of artificial trees (%)	40.00 ± 4.63c	75.00 ± 2.45a	50.00 ± 2.45b	–
Average diameter at breast height (cm) of artificial trees	17.58 ± 0.81c	27.03 ± 2.51a	22.67 ± 1.11b	–
Average tree height of artificial trees (m)	16.66 ± 1.58b	15.90 ± 1.41b	21.73 ± 0.91a	–
Average branch height (m) of artificial trees	12.98 ± 0.41b	6.73 ± 1.14c	14.89 ± 0.51a	–
Initial plant number density of artificial trees (plants/ha)	1667	800	1667	–
Density of existing artificial forest trees (plants/ha)	800.00 ± 32.66a	625.00 ± 18.71b	341.67 ± 58.93c	–
Average diameter at breast height (cm) of natural regeneration trees	6.62 ± 0.68b	4.25 ± 0.12c	6.40 ± 0.93b	8.20 ± 0.54a
Average height of natural regeneration trees (m)	5.98 ± 0.26b	4.16 ± 0.09c	7.02 ± 1.12a	7.32 ± 0.19a
Natural regeneration tree number density (plants/ha)	3533.33 ± 249.44a	1804.17 ± 163.41c	1066.67 ± 135.91d	2983.33 ± 185.22b

AM, HB, ER, and SF respectively represent *Acacia mangium* forest, *Hevea brasiliensis* forest, *Eucalyptus* forest, and natural secondary forest. Different lowercase letters indicate significant differences between stands (P < 0.05).

### 3.2 Comparison of differences in community structure among different forest stands

#### 3.2.1 Comparison of differences in the structure of the arbor regeneration layer

**Figure 2:**
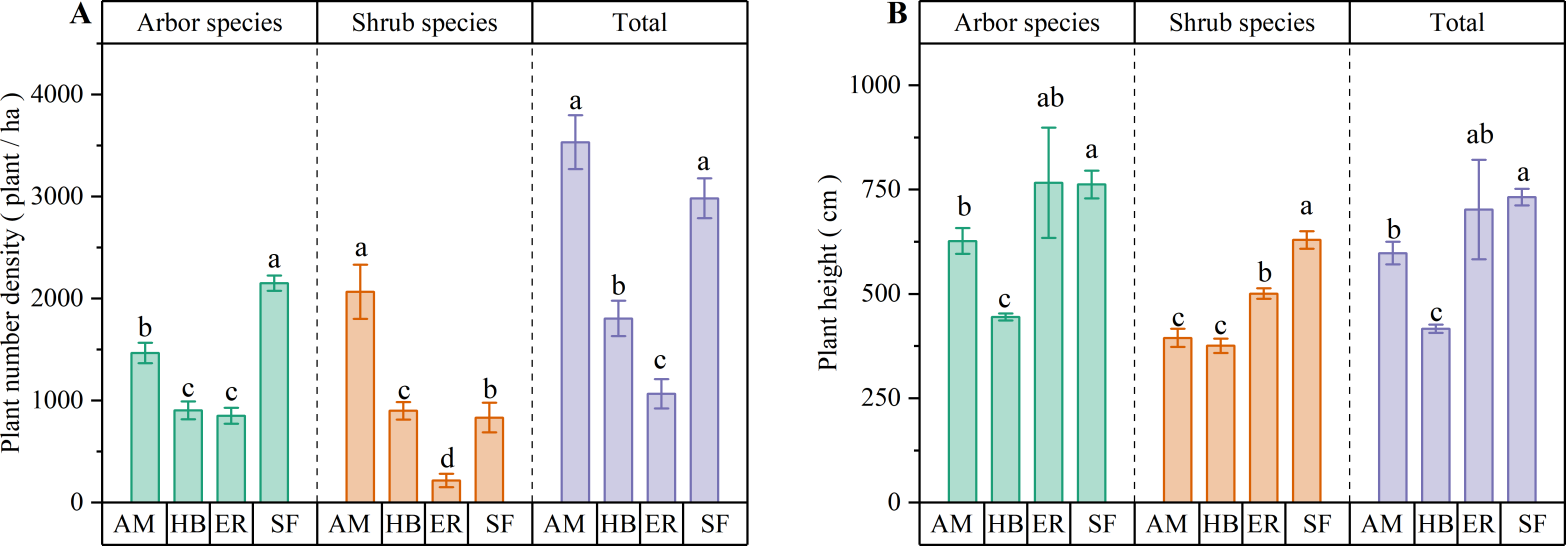
Number density and average height of arbor layer in different regeneration layers of artificial forest and natural secondary forest. **(A)** Plant number density; **(B)** Average plant height. AM, HB, ER, and SF respectively represent *Acacia mangium* forest, *Hevea brasiliensis* forest, *Eucalyptus* forest, and natural secondary forest. Different lowercase letters indicate significant differences between stands (P < 0.05).

As shown in **Figure 2**, the AM forests exhibited significantly higher (P < 0.05) arbor and shrub species densities than both the HB and ER forests. Although arbor species density did not differ significantly between the HB and ER forests, shrub species density was markedly higher (P < 0.05) in the former. All three plantation types demonstrated significantly lower (P < 0.05) arbor species densities than the natural secondary forest. Notably, the AM forests had a significantly higher shrub species density than the natural secondary forests (P < 0.05). These results suggested that the AM forests supported greater woody plant regeneration, possibly because of a more open canopy structure. The consistently reduced arbor species density in all plantation types compared with the natural forest underscored the inherent limitations in plantation ecosystem recovery.

Among the three plantation types, the ER forests exhibited the greatest mean heights for both arbor and shrub species. The arbor species height in the ER forests was similar to that in the AM forests but significantly higher (P < 0.05) than in the HB forests, while the shrub species height was significantly greater (P < 0.05) than in the other two plantations. Compared with the natural secondary forest, the arbor species height in the ER forests was not significantly different, whereas the shrub species were significantly shorter (P < 0.05). In contrast, both arbor and shrub species in the AM and HB forests were significantly shorter (P < 0.05) than those in the natural secondary forest. These findings suggested that tree height development in the ER forests approximated that of natural secondary forests, which could be due to the greater branch height of the artificial trees. This provides more vertical space for regenerating species. However, the consistently shorter woody plant heights in the AM and HB forests indicated limited regeneration potential in the arbor layer.

As shown in **Table 3**, distinct patterns in diameter class diversity indices were observed among the forest stands. The DBH-Shannon index followed the order AM > ER > HB, whereas the DBH-Simpson index ranked ER > AM > HB. Although no significant differences (P > 0.05) were detected between the ER and AM forests for either index, both exhibited significantly higher values (P < 0.05) than those of the HB forests. The DBH-Pielou index ranked ER > AM > HB, with no significant differences (P > 0.05) among the plantation types. All three plantations displayed significantly lower (P < 0.05) diameter class diversity indices than the natural secondary forests. These findings suggested that after two decades of natural recovery, the ER and AM forests developed greater diameter class heterogeneity than the HB forests. The reduced diversity in the HB forests may result from denser artificial canopies restricting natural regeneration. Nonetheless, none of the plantations reached the diameter class diversity observed in natural secondary forests, highlighting the structural limitations of plantation systems in replicating the complexity of natural forests.

**Table 3:** Diameter class diversity index of the arbor regeneration layer of different plantations and arbor layers of natural secondary forests.

Indicator	Forest stand type			
	AM	HB	ER	SF
DBH-Shannon	2.19 ± 0.08b	1.34 ± 0.16c	2.00 ± 0.24b	2.58 ± 0.16a
DBH-Simpson	0.79 ± 0.04b	0.67 ± 0.10c	0.81 ± 0.02b	0.89 ± 0.03a
DBH-Pielou	0.71 ± 0.02b	0.69 ± 0.02b	0.74 ± 0.03b	0.79 ± 0.02a

AM, HB, ER, and SF respectively represent *Acacia mangium* forest, *Hevea brasiliensis* forest, *Eucalyptus* forest, and natural secondary forest. Different lowercase letters indicate significant differences between stands (P < 0.05).

#### 3.2.2 Comparison of differences in shrub layer structure

**Figure 3:**
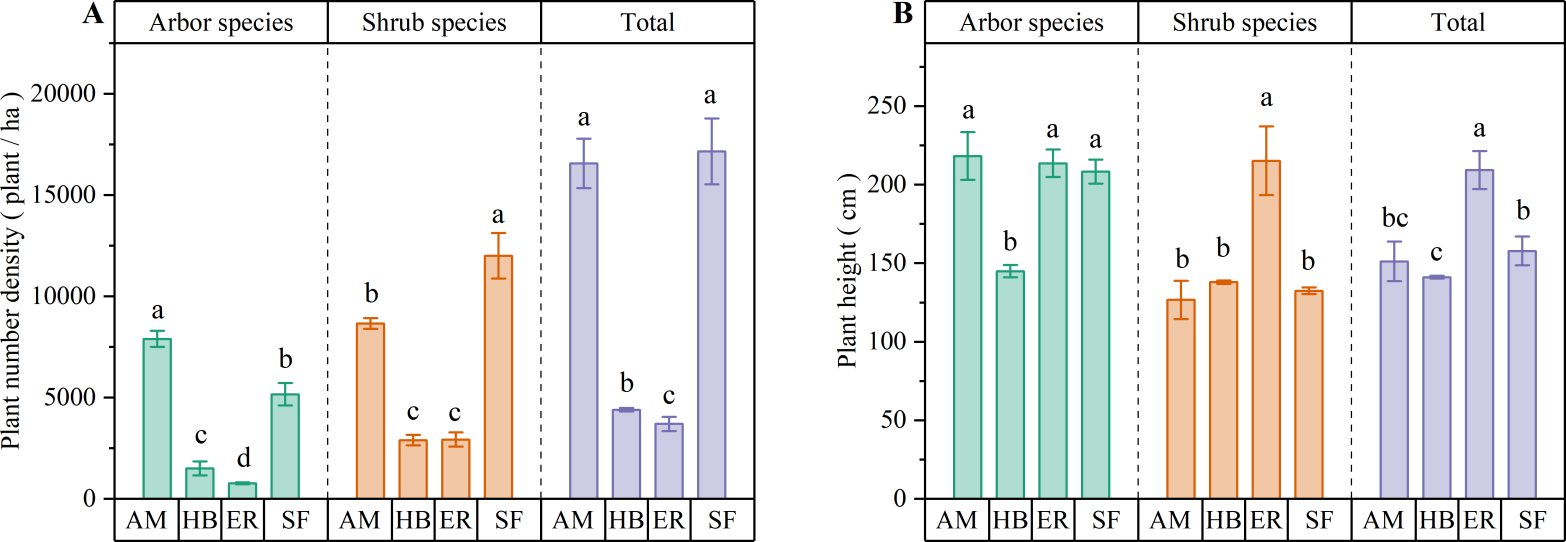
Number density and average plant height of shrub layers in different forest stands. **(A)** Plant number density; **(B)** Average plant height. AM, HB, ER, and SF respectively represent *Acacia mangium* forest, *Hevea brasiliensis* forest, *Eucalyptus* forest, and natural secondary forest. Different lowercase letters indicate significant differences between stands (P < 0.05).

As shown in **Figure 3**, the AM forests exhibited significantly higher densities of arbor, shrub, and total individuals in the shrub layer than the HB and ER forests (P < 0.05). The HB forests had greater arbor species and total densities than the ER forests (P < 0.05), whereas the shrub species densities did not differ significantly between the two (P > 0.05). Notably, the AM forests surpassed the natural secondary forests in the arbor species density (P < 0.05), while no significant difference in the total density was observed (P > 0.05). In contrast, the HB and ER forests exhibited significantly lower densities of arbor, shrub, and total individuals than the natural secondary forests (P < 0.05). These results indicated that the AM forests demonstrated superior shrub layer regeneration capacity, particularly for arbor species, even exceeding the natural secondary forests in arbor recruitment. In contrast, shrub layer regeneration in the HB and ER forests appeared constrained, which could be due to the higher herb layer coverage, highlighting their limited capacity for spontaneous woody plant establishment.

Among the three plantation types, the AM forests contained the tallest arbor species in the shrub layer, with heights similar to those in the ER forests (P > 0.05) but significantly greater than those in the HB forests (P < 0.05). Conversely, the ER forests exhibited significantly greater shrub species and total shrub layer heights than both the AM and HB forests (P < 0.05), whereas the latter two showed no significant differences (P > 0.05). Compared with the natural secondary forest, the AM forests demonstrated equivalent heights for arbor species, shrub species, and the overall shrub layer (P > 0.05). The ER forests matched the natural forest in arbor species height (P > 0.05) but significantly exceeded it in the shrub species and the total heights (P < 0.05). The HB forests displayed significantly shorter arbor species and total shrub layer heights than the natural secondary forest (P < 0.05), whereas the shrub species height remained comparable (P > 0.05). These results suggest that ER forests promote pronounced vertical growth within the shrub layer, potentially driven by light competition under dense herbaceous cover. The shrub layer height structure in the AM forests most closely resembled natural forest conditions, reflecting distinct ecological dynamics among the forest types.

#### 3.2.3 Comparison of differences in herb layer structure

**Figure 4:**
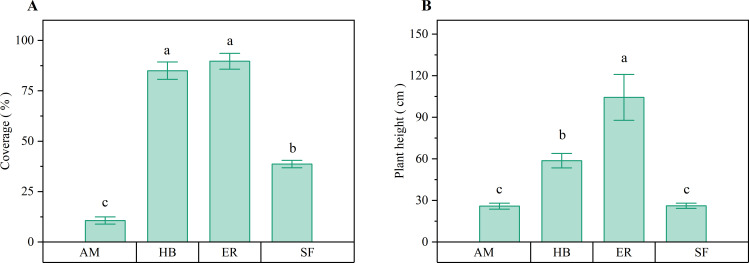
Herb layer coverage and average plant height of different forest stands. **(A)** Coverage; **(B)** Average plant height. AM, HB, ER, and SF respectively represent *Acacia mangium* forest, *Hevea brasiliensis* forest, *Eucalyptus* forest, and natural secondary forest. Different lowercase letters indicate significant differences between stands (P < 0.05).

**Figure 4** presents the observation of distinct patterns in herb layer development across forest stands. Herb layer coverage followed the order ER > HB > AM, with the ER and HB forests exhibiting similar coverage (P > 0.05), both significantly higher than that of AM forests (P < 0.05). Compared with natural secondary forests, herb coverage increased by 51.00% and 46.33% in the ER and HB forests, respectively (P < 0.05), whereas it decreased by 28.00% in the AM forests (P < 0.05). Herb layer height followed a similar trend (ER > HB > AM), with all inter-plantation differences being significant (P < 0.05). The ER and HB forests exceeded the natural secondary forest height by 78.20 and 32.54 cm, respectively (P < 0.05), whereas the AM forests showed no significant difference in height (P > 0.05). These results indicated that after two decades of recovery, the ER and HB forests developed significantly denser and taller herb layers than both the AM forests and natural secondary forests. This pattern could be attributed to reduced woody plant competition, which increased light availability and promoted herbaceous growth.

### 3.3 Comparison of differences in plant diversity among different forest stands

#### 3.3.1 Comparison of differences in arbor layer plant diversity

**Figure 5:**
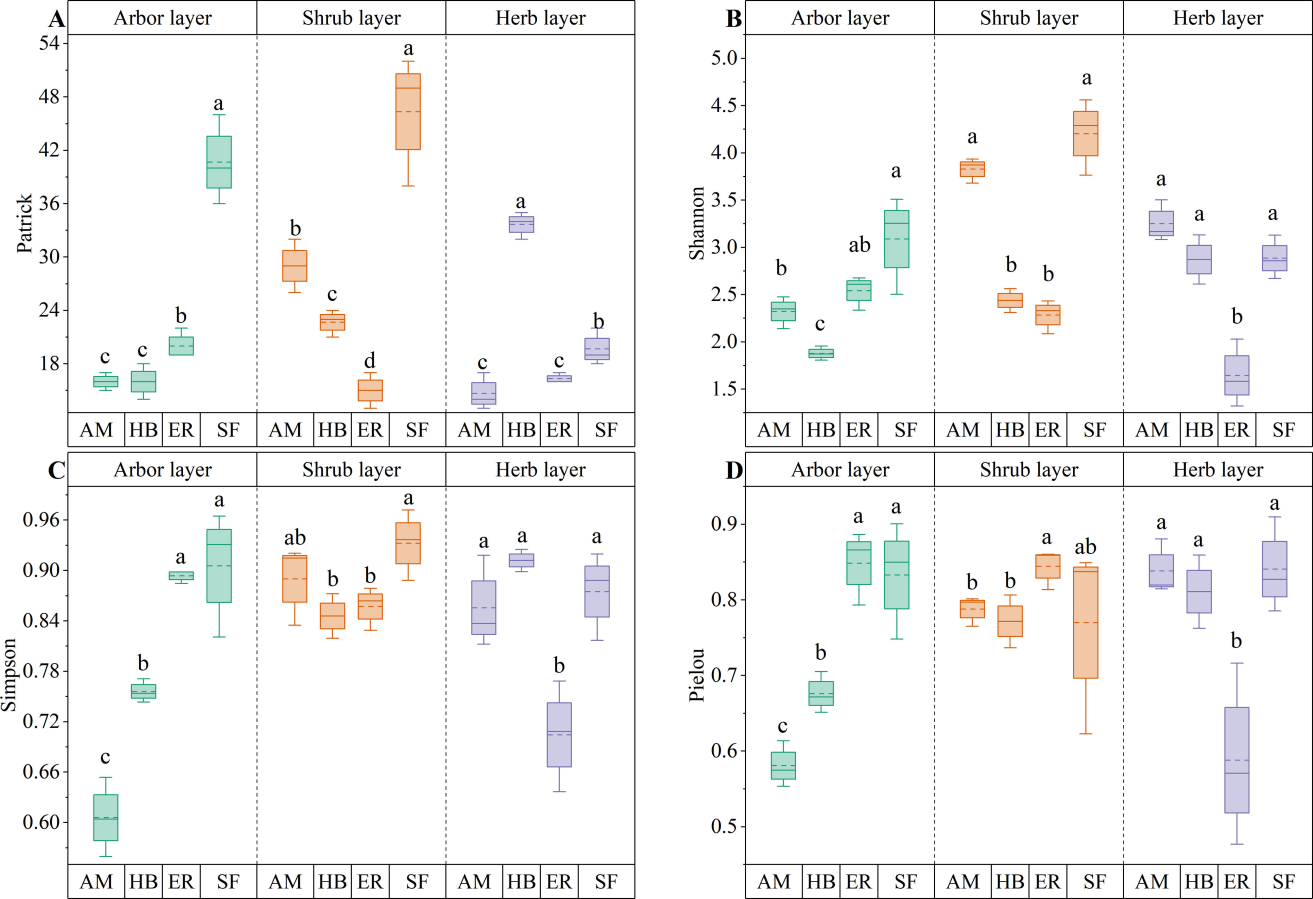
Plant species diversity indices for different forest stands. **(A)** Patrick index; **(B)** Shannon index; **(C)** Simpson index; **(D)** Pielou index. AM, HB, ER, and SF respectively represent *Acacia mangium* forest, *Hevea brasiliensis* forest, *Eucalyptus* forest, and natural secondary forest. Different lowercase letters indicate significant differences between stands (P < 0.05).

As shown in **Figure 5**, distinct patterns in arbor layer species diversity were observed among the forest stands. The Patrick and Shannon indices followed the order ER > AM > HB, with the ER forests exhibiting significantly higher Patrick values than the AM and HB forests (P < 0.05). Although the Shannon indices were comparable between the ER and AM forests (P > 0.05), both significantly exceeded those of the HB forests (P < 0.05). Simpson and Pielou indices ranked ER > HB > AM, with all pairwise differences being significant (P < 0.05). Compared with the natural secondary forests, the ER forests achieved similar Shannon, Simpson, and Pielou index values (P > 0.05), although their Patrick indices remained significantly lower (P < 0.05). In contrast, both AM and HB forests showed significantly lower values across all diversity indices (P < 0.05). These results suggested that after two decades of natural recovery, the ER forests have developed arbor layer species diversity approaching that of natural secondary forests, significantly exceeding that of the AM and HB forests. This pattern reflected divergent successional trajectories among different forest types, with the ER forests exhibiting the greatest potential for species diversity recovery in the tree layer.

**Figure 6:**
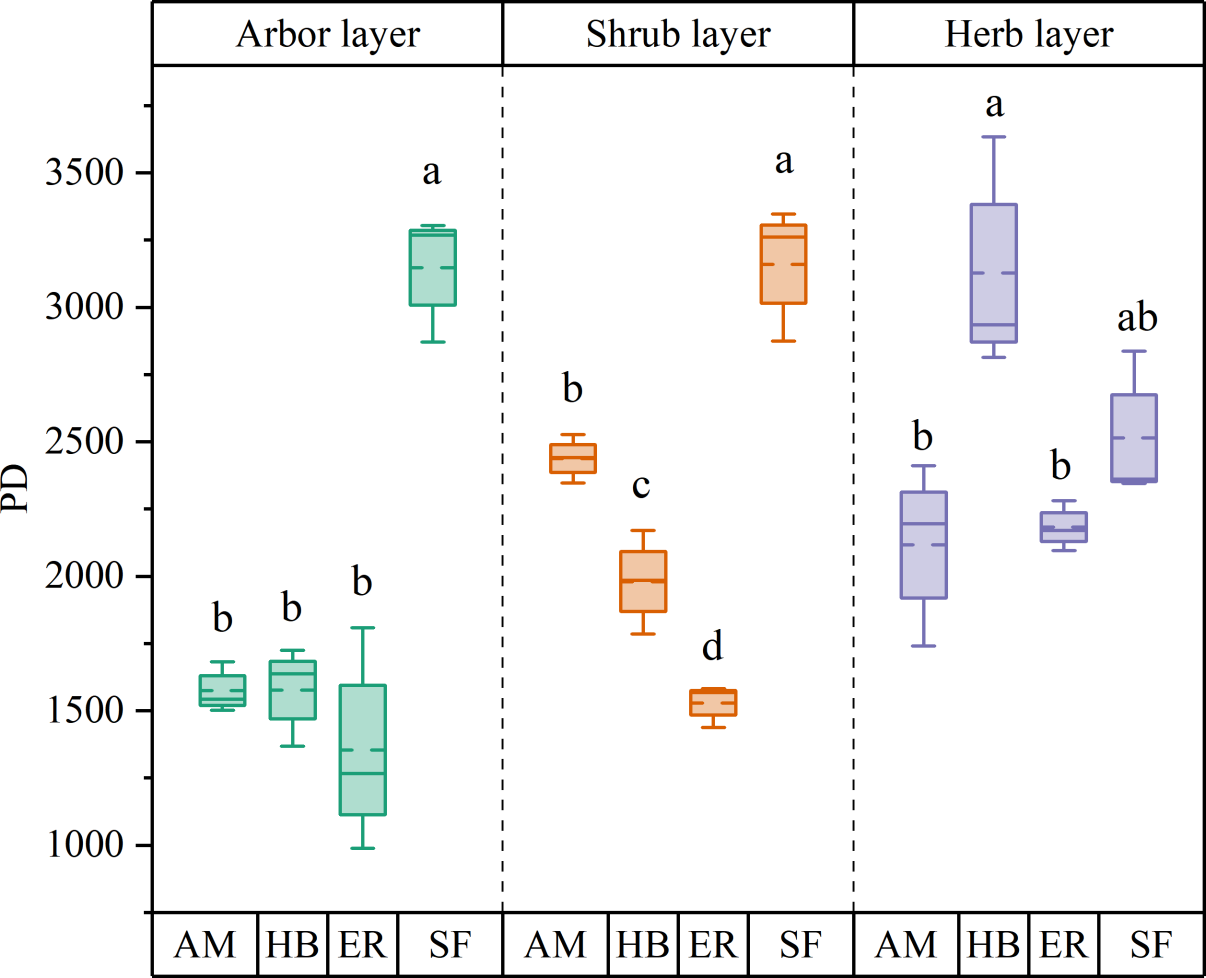
α diversity index of plant phylogeny in different stands. AM, HB, ER, and SF respectively represent *Acacia mangium* forest, *Hevea brasiliensis* forest, *Eucalyptus* forest, and natural secondary forest. Different lowercase letters indicate significant differences between stands (P < 0.05).

**Figure 7:**
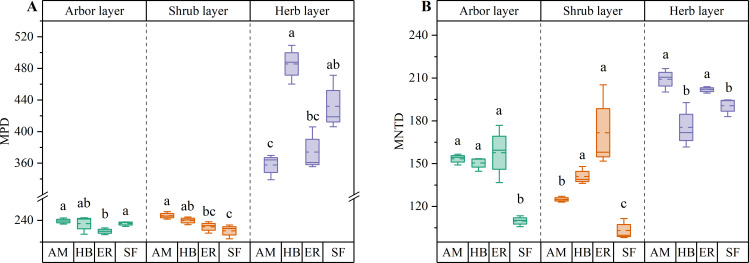
β diversity index of plant phylogeny in different stands. **(A)** MPD; **(B)** MNTD. AM, HB, ER, and SF respectively represent *Acacia mangium* forest, *Hevea brasiliensis* forest, *Eucalyptus* forest, and natural secondary forest. Different lowercase letters indicate significant differences between stands (P < 0.05).

PD in the arbor layer followed the order HB > AM > ER, with no significant differences among the three plantation types (P > 0.05). MPD ranked AM > HB > ER, with AM forests exhibiting a significantly higher MPD than ER forests (P < 0.05), whereas HB forests did not differ significantly from either (P > 0.05). In contrast, MNTD followed the sequence ER > AM > HB, with no significant differences observed among the forests (P > 0.05). All three forests showed significantly lower PD than the natural secondary forests (P < 0.05). Notably, MPD in the AM and HB forests did not differ significantly from that in the natural forests (P > 0.05), whereas the ER forests exhibited significantly lower MPD (P < 0.05). However, MNTD was significantly higher in all forests than in natural secondary forests (P < 0.05) (**Figures 6**, **7**). These findings indicated that the three plantations exhibited comparable but significantly lower PD than the natural forests after the 20-year restoration. Moreover, the higher phylogenetic β diversity suggested increased phylogenetic dispersion, whereas none of the plantations replicated the phylogenetic structure characteristics of natural secondary forests.

#### 3.3.2 Comparison of differences in shrub layer plant diversity

As shown in **Figure 5**, distinct patterns in shrub layer species diversity were observed among forest stands. Both the Patrick and Shannon indices followed the order AM > HB > ER, with significant differences in the Patrick index across all forest types (P < 0.05). The Shannon index in the AM forests was significantly higher than that in the HB and ER forests (P < 0.05). The Pielou index followed the order ER > AM > HB, with the ER forests significantly exceeding both the HB and AM forests (P < 0.05), whereas the Simpson index showed no significant differences among the forests (P > 0.05). Compared with the natural secondary forests, only the Patrick index in the AM forests was significantly lower (P < 0.05), whereas the Shannon, Simpson, and Pielou indices were comparable (P > 0.05). In contrast, HB and ER forests exhibited significantly lower values across the Patrick, Shannon, and Simpson diversity indices (P < 0.05). These results indicated that the shrub layer species diversity in the AM forests exceeded that of the HB and ER forests and closely approached the diversity levels observed in the natural secondary forests.

Both PD and MPD followed the order AM > HB > ER, with the AM forests exhibiting a significantly higher PD than the other forests (P < 0.05) and a significantly greater MPD than the ER forests (P < 0.05). In contrast, MNTD showed an inverse pattern (ER > HB > AM), with the AM forests having significantly lower values than the other two types (P < 0.05). Compared to the natural secondary forests, all forests displayed significantly lower PD (P < 0.05) and higher MNTD (P < 0.05), indicating increased phylogenetic dispersion. Although the AM and HB forests exhibited significantly higher MPD than the natural forests (P < 0.05), the ER forests showed no significant difference (P > 0.05) (**Figures 6**, **7**). These findings indicated that the phylogenetic α diversity in the shrub layer of the AM forests was higher than that in other types, with the species more closely related phylogenetically. Although the PD of all three plantations was significantly lower than that of the natural secondary forests, their phylogenetic dispersion was comparatively greater.

#### 3.3.3 Comparison of differences in herb layer plant diversity

As shown in **Figure 5**, distinct patterns in herb layer species diversity were observed among forest stands. The Patrick index followed the order HB > ER > AM, with HB forests exhibiting significantly higher values than the other forest types (P < 0.05). In contrast, the Shannon and Pielou indices followed the order AM > HB > ER, with the AM and HB forests showing statistically similar values (P > 0.05), both of which significantly exceeded those of the ER forests (P < 0.05). The Simpson index followed HB > AM > ER, with no significant difference between the top two (P > 0.05), both of which were significantly higher than those in ER forests (P < 0.05). Compared to the natural secondary forests, the HB forests had significantly higher Patrick index values (P < 0.05), whereas the AM forests exhibited significantly lower values (P < 0.05). However, both maintained Shannon, Simpson, and Pielou indices similar to those of the natural secondary forests (P > 0.05). In contrast, the ER forests exhibited significantly lower values for all diversity indices (P < 0.05). These findings indicated that the herb layer species richness in the HB forests was significantly higher than that in the other two plantations. Apart from species richness, the remaining diversity indices in the herb layers of the HB and AM forests were comparable to those of the natural secondary forests.

HB forests exhibited the highest PD and MPD, significantly exceeding those of the ER and AM forests (P < 0.05). In contrast, MNTD followed an inverse pattern, with the AM and ER forests displaying statistically comparable values (P > 0.05), both of which were significantly higher than those of the HB forests (P < 0.05). Comparative analysis of natural secondary forests revealed no significant differences in PD across forest types (P > 0.05). Although the AM forests showed significantly lower MPD than the natural secondary forests (P < 0.05), the HB and ER forests maintained similar MPD levels (P > 0.05). Regarding MNTD, the AM and ER forests exhibited significantly higher values than the natural forests (P < 0.05), whereas the HB forests showed no significant difference (P > 0.05) (**Figures 6**, **7**). These findings indicated that the herb layer PD and phylogenetic breadth in the HB forests were greater than those in the other two forests and closely resembled those of the natural secondary forests. However, lower MNTD values suggested a higher degree of clustering in the nearest phylogenetic relationships among species.

### 3.4 Relationship between plant diversity and community structure factors in plantations

#### 3.4.1 Relationship between arbor layer plant diversity and community structure factors

**Figure 8:**
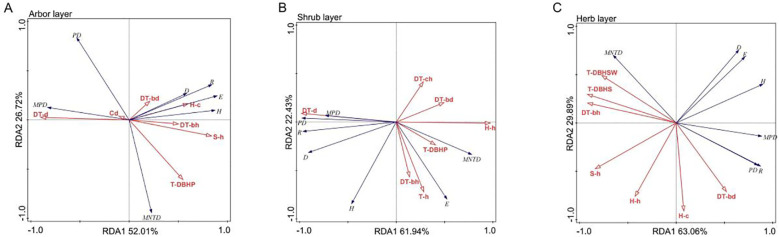
RDA ordination diagram of plant diversity and community structure factors in each layer of the artificial forest. **(A)** Arbor layer; **(B)** Shrub layer; **(C)** Herb layer. R, H, D, E, PD, MPD, and MNTD represent Patrick index, Shannon index, Simpson index, Pielou index, PD, MPD, and MNTD, respectively. DT-d, DT-ch, DT-bh, DT-h, DT-bd, Cd, T-d, T-h, T-bd, S-d, S-h, H-c, H-h, T-DBHSW, T-DBHS, and T-DBHP represent the number density of artificial trees, the average crown width of artificial trees, the average branch height of artificial trees, the height of artificial trees, the average DBH of artificial trees, the canopy coverage of artificial trees, the number density of arbor regeneration layer, the average height of arbor regeneration layer, the average DBH of arbor regeneration layer, the number density of shrub layer, the average height of shrub layer, the coverage of herbs, the average height of herb layer, DBH-Shannon index, DBH-Simpson index, and DBH-Pie index.

The first two axes of the RDA jointly explained 78.73% of the total variation (RDA1 = 52.01% and RDA2 = 26.72%), with the number density of artificial trees (43.2% explained variance), DBH-Pielou index (16.6%), and branch height of artificial trees (14.9%) identified as the key driving factors (**Tables 4**, **5**). Species diversity indices (Patrick, Shannon, Simpson, and Pielou) were positively correlated with branch height and DBH of artificial trees, herb layer cover, and shrub layer height and negatively correlated with the number density and canopy cover of artificial trees. In contrast, MPD and PD were positively associated with the number density and canopy cover of artificial trees (**Figure 8A**). These results suggested that higher artificial tree density and canopy cover suppressed species diversity in the arbor layer while promoting phylogenetic dispersion.

**Table 4:** RDA of plant diversity and community structure factors in different layers of artificial forests.

Statistical value	Arbor layer		Shrub layer		Herb layer	
	RDA1	RDA2	RDA1	RDA2	RDA1	RDA2
Eigenvalues	0.5201	0.2672	0.6194	0.2243	0.6306	0.2989
Cumulative explainable variation (%)	52.01	78.73	61.94	84.36	63.06	92.95
Correlation coefficient between plant diversity and community structure factors	0.9993	0.9965	0.9999	0.9907	0.9999	0.9985

**Table 5:** RDA interpretation and significance test of plant diversity and community structure factors in different layers of artificial forests.

Forest layer	Structural factor	Factor interpretation (%)	*F*	*P*
Arbor layer	Number density of artificial trees	43.2	5.3	0.006
	DBH-Pielou index	16.6	2.5	0.048
	Average branch height of artificial trees	14.9	2.9	0.046
	Coverage of herbs	9.9	2.6	0.092
	Canopy coverage of artificial trees	8.1	7.4	0.010
	Average height of shrub	2.0	1.6	0.262
	Average DBH of artificial trees	1.6	1.8	0.334
shrub layer	Number density of artificial trees	59.7	10.4	0.002
	Average height of arbor regeneration layer	15.2	3.6	0.050
	Average crown width of artificial trees	9.2	4.1	0.022
	Average height of herb layer	6.8	1.8	0.174
	Average DBH of artificial trees	5.0	3.7	0.060
	DBH-Pielou index	1.8	1.6	0.340
	Average branch height of artificial trees	1.4	1.7	0.386
Herb layer	DBH-Simpson index	55.7	8.8	0.002
	Average height of shrub layer	30.4	13.1	0.002
	Average DBH of artificial trees	3.1	1.4	0.276
	Coverage of herbs	2.8	1.4	0.270
	Average height of herb layer	2.7	1.6	0.258
	DBH-Shannon index	2.8	2.2	0.212
	Average branch height of artificial trees	1.9	3.2	0.276

#### 3.4.2 Relationship between shrub layer plant diversity and community structure factors

The first two RDA axes jointly explained 84.36% of the total variation (RDA1 = 61.94% and RDA2 = 22.43%), with the number density of artificial trees (59.7%) and the height of the arbor regeneration layer (15.2%) identified as the key driving factors (**Tables 4**, **5**). MPD, PD, and species diversity indices (except the Pielou index) were positively correlated with the number density of artificial trees but negatively correlated with the DBH of artificial trees, mean height of the arbor regeneration layer, DBH-Pielou index, and herb layer height. In contrast, the Pielou index and MNTD showed positive associations with the herb layer height and height of the arbor regeneration layer (**Figure 8B**). These results indicated that a high density of artificial trees promoted species settlement in the shrub layer, enhancing plant diversity, while the increased height in the arbor regeneration and shrub layers may suppress the shrub layer diversity through resource competition.

#### 3.4.3 Relationship between plant diversity in the herb layer and community structural factors

The first two RDA axes explained a cumulative 92.95% of the total variation (RDA1 = 63.06%, RDA2 = 29.89%), with the DBH-Simpson index (55.7%) and shrub layer height (30.4%) identified as the key driving factors (**Tables 4**, **5**). Simpson, Shannon, and Pielou indices were negatively correlated with the average height of the shrub and herb layers. The PD, MPD, and Patrick indices were negatively associated with the DBH-Shannon index, DBH-Simpson index, and mean branch height of artificial trees but positively correlated with the average DBH of artificial trees and herb layer coverage (**Figure 8C**). These results suggest that the increased diameter class differentiation in the arbor regeneration layer along with the greater shrub and herb layer heights may suppress plant diversity in the herb layer.

## 4 Discussion

### 4.1 Characteristics of community structure in different forest stands

The growth status of forest stands serves as a key indicator for assessing the transition from plantations to natural forests because it directly influences the trajectory and ecological feasibility of this transformation (Wang et al., 2021). In this study, substantial variability in the growth parameters of artificial trees (crown coverage, mean DBH, tree height, and branch height) was observed among forest types, which could be attributed to species-specific biological traits and ecological adaptations (Friedrichs et al., 2009). The ER and AM forests exhibited the greatest reductions in artificial tree density (79.50% and 52.00%, respectively), likely resulting from the initially high planting densities that induced negative density-dependent mortality (Wright, 2002). Both species are characterized by rapid growth and short rotation cycles, contributing to age-related senescence and mortality in later stages (Laclau et al., 2013). Significant differences were also evident in the growth performance of naturally regenerated trees. The AM forests supported the highest mean DBH and density of naturally regenerated trees, potentially because of their sparse canopy structure, which enhanced light availability and created favorable microsites for sapling establishment (He et al., 2023; Wagner et al., 2011). Additionally, the nitrogen-fixing capacity of AM improves soil fertility, supporting the coexistence and survival of associated woody species (Wu et al., 2024). In contrast, the ER forests produced the tallest naturally regenerated trees, with heights comparable to those in natural secondary forests, likely because of a relatively open stand structure that reduced light competition (Galván-Cisneros et al., 2023). Nevertheless, the naturally regenerated trees in all three plantations exhibited significantly lower densities and mean DBH than those in the natural secondary forests, indicating consistent limitations in growth under passive restoration (Asanok et al., 2013). These findings highlight the need to optimize stand structural complexity and refine silvicultural strategies to improve natural regeneration during plantation succession.

Understory community structure is a key component of forest ecosystems, influencing ecological functions, biodiversity, and resilience potential (Bricca et al., 2023; Deng et al., 2023). In this study, the AM forests exhibited the highest individual densities in both arbor regeneration and shrub layers, indicating a strong regeneration capacity during forest restoration. The diameter diversity index further supported the superior natural regeneration performance of AM forests. In contrast, HB and ER forests showed markedly lower individual densities in the shrub layer, accounting for only 25.63% and 21.55% of those in the natural secondary forests, respectively. This may be attributed to the high coverage and prominent average height of the herb layer in these forests, which occupied critical ecological niches and inhibited woody plant establishment in the shrub layer. Nevertheless, all three plantation types demonstrated lower arbor species densities in the arbor regeneration layer than in the natural secondary forests, which could be due to the relatively short restoration period and suboptimal growth conditions within the plantations (Wang et al., 2009; Asanok et al., 2013; Puchałka and Płąchocki, 2014).

### 4.2 Plant diversity in different forest stands

Plant species diversity is a key indicator of forest ecosystem health and the effectiveness of ecological restoration efforts (Poorter et al., 2021). The results of this study indicated that the arbor layer in the ER forests exhibited the highest species diversity among the artificial forest types, likely due to reduced tree density and enhanced understory light availability resulting from a sparse canopy. Traditionally, ER has been regarded as a tree species with a relatively large impact on the ecological environment (Ping et al., 2009; Galván-Cisneros et al., 2023). However, this study revealed that under natural restoration, ER forests demonstrated high potential for the restoration of species diversity in the tree layer. These findings may help shift traditional perceptions and highlight the ecological benefits of ER forests. In contrast, the HB forests displayed lower arbor layer diversity, which was attributed to dense crown cover. In the shrub layer, the AM forests demonstrated superior species diversity compared to other types, with no significant differences from the natural secondary forests in all indices except the Patrick index. This suggested that AM forests effectively restored woody plant diversity to levels approaching those of natural secondary forests, which is consistent with the findings of Peng et al. (2015). However, both the arbor and shrub layers of all forests exhibited lower species richness than natural secondary forests, likely because of competitive exclusion and habitat fragmentation during the restoration process (Shan, 2021). The Patrick index of the herbaceous layer in HB forests was significantly higher than that in the other two plantation types and natural secondary forests. This may be attributed to the periodic “gap effect” formed by the seasonal leaf-fall characteristic of HB forests (Xue et al., 2022), which significantly increases understory light, provides diverse niches for herbaceous plants, and promotes the colonization of more herbaceous plant species. Notably, the herb layers in the HB and AM forests showed no significant differences from the natural secondary forests in the Shannon, Simpson, and Pielou indices, indicating comparable diversity and evenness. In contrast, the ER forests supported a monodominant herbaceous community dominated by pioneer species (e.g., *Dicranopteris dichotoma*), which suppressed the establishment and spread of other herbaceous taxa, thereby reducing overall diversity. From the perspective of long-term woody plant restoration, the dominance of a single herbaceous species may occupy substantial ecological resources such as light, water, and nutrients, thereby restricting the growth and survival of woody seedlings and delaying the successional transition from plantations to natural forests (Shen and Cao, 2010). However, short-term herbaceous dominance is not entirely detrimental. In the early stages of restoration, herbaceous plants can rapidly establish ground cover, reduce soil erosion, improve soil structure, and create favorable conditions for the subsequent invasion and growth of woody plants (Yang et al., 2018). Therefore, effective tropical plantation restoration requires a comprehensive consideration of both short- and long-term ecological goals. It is essential to strike a balance between herbaceous plant dominance and woody plant regeneration and to formulate scientific and rational restoration strategies.

This study demonstrated that after 20 years of natural restoration, the PD of arbor and shrub layers in all artificial forests remained significantly lower than that of natural secondary forests, indicating incomplete convergence toward the evolutionary characteristics of natural systems. This finding highlights the inherent limitations of woody plant restoration in artificial ecosystems. Moreover, the artificial forests exhibited a higher MNTD in both arbor and shrub layers, likely resulting from intensified competitive exclusion during early restoration stages and pronounced environmental shifts throughout succession (Galván-Cisneros et al., 2023; Huang et al., 2023). In the shrub layer, the AM forests exhibited significantly higher PD than the other artificial forests, which was attributed to greater species richness. In contrast, the ER and HB forests displayed an elevated shrub-layer MNTD compared to the AM and natural secondary forests, potentially reflecting niche differentiation driven by herb layer dominance. In these systems, vigorous herbaceous growth may constrain shrub-layer woody species to diverge phylogenetically to minimize competitive overlap (Backhaus et al., 2021). Within the herb layer, the HB forests exhibited the highest PD and MPD but lower MNTD than the ER and AM forests. This pattern is consistent with environmental filtering effects (Du et al., 2025). The dense canopy cover in the HB forests produced a homogenized shaded microenvironment that favored phylogenetically conserved lineages with convergent ecological traits. In contrast, the sparser canopies of the ER and AM forests enhance light heterogeneity in the understory, facilitating the coexistence of distantly related herbaceous species (Stein et al., 2014; Wills et al., 2021).

### 4.3 Relationship between plant diversity and community structure factors in artificial forests

Elucidating the relationship between plant diversity and community structural factors in artificial forests at a regional scale provides critical insights for informing scientific management and guiding the ecological restoration of natural forest analogs (Deng et al., 2022; Li et al., 2025a). The RDA results identified artificial tree density, DBH-Pielou index, and average branch height as the key drivers of arbor layer plant diversity under natural restoration. Specifically, reduced artificial tree density and increased branch height enhanced horizontal and vertical spatial availability, facilitating the recruitment of new arbor species. Higher DBH-Pielou values, indicating a more uniform diameter distribution, were associated with increased plant diversity, which is consistent with the findings of Kang et al. (2016). The density and average height of the arbor regeneration layer were the primary determinants of shrub layer diversity, underscoring the regulatory role of the overstory structure in shaping understory dynamics. The shrub layer species diversity, PD, and MPD were negatively correlated with herb layer height, suggesting competitive suppression by the dominant herbaceous species (Yang et al., 2017). The DBH-Simpson index and average shrub height were identified as the key drivers of herb layer diversity, both showing negative correlations with most herb layer diversity indices (except MNTD), likely due to the limitations in resource availability (e.g., light, water, and nutrients) imposed by the upper-layer structure. Moreover, herb layer height was negatively associated with several diversity metrics, reflecting the potential competitive exclusion by dominant tall herbs that restricted the establishment and reproduction of subordinate herbaceous species. According to the above findings, it is necessary to reduce the stem density and canopy coverage of artificial trees through selective thinning. Additionally, removing certain herbaceous plants that occupy dominant ecological niches can help release understory space, alleviate resource competition and photothermal inhibition, and ultimately promote overall restoration of plant diversity in plantations. Moreover, the restoration of the tree layer in the three plantation types was relatively slow under natural restoration and faced certain limitations. To enhance tree layer regeneration, it may be beneficial to supplement natural restoration with strategic introduction of native broad-leaved tree species into each plantation.

Despite the meaningful findings of this study, it has several limitations. First, forest restoration is a long-term process, and the 20-year restoration period examined for the three plantation types was relatively short. Second, the study was conducted at a single site, the Fengmu Experimental Forest Farm, which may limit the generalizability of the results to this region or areas with similar ecological conditions. Future studies should consider extending the restoration period and expanding the geographical scope to gain a more comprehensive understanding of the ecological mechanisms involved in tropical plantation restoration.

## 5 Conclusion

After 20 years of natural recovery, the AM, HB, and ER forests developed into multi-layered, mixed, and uneven-aged forests with distinct arbor-shrub-herb stratification. Notably, AM forests exhibited the highest potential for natural regeneration, whereas limitations persisted in the growth of naturally regenerated trees during the restoration process. The ER, AM, and HB forests demonstrated the highest species diversity in the arbor, shrub, and herb layers, respectively, approaching the diversity levels observed in the corresponding layers of natural secondary forests. However, the woody plants in all three plantation types could not attain the phylogenetic characteristics of natural secondary forests, with relatively large phylogenetic distances between species. Plant diversity in plantations was closely associated with community structure attributes. High artificial tree density and canopy coverage suppressed arbor layer species diversity but promoted phylogenetic divergence. While dense artificial tree stands facilitated shrub layer colonization, vertical growth in the arbor regeneration and shrub layers suppressed shrub layer diversity through resource competition. The diameter class differentiation in the arbor regeneration layer and the increased shrub-herb layer height reduced herb layer diversity owing to resource limitations. These findings indicated that naturally recovering plantations still exhibited certain constraints in the development of the arbor layer and overall biodiversity restoration. Therefore, optimizing and regulating stand structure is essential for promoting niche differentiation and accelerating succession in climax forest communities.

## Data Availability

The original contributions presented in the study are included in the article/supplementary material. Further inquiries can be directed to the corresponding author.
